# 

**Published:** 1917-05-12

**Authors:** 


					HOSPITAL AND INSTITUTIONAL RESULTS.
Royal West Sussex Hospital, Chichester.?The
average cost per occupied bed in 1916 was ?75 9s. 3d.
The Ladies' Linen. League contributed 1,545 articles.
Total income, ?6,575; total expenditure, ?5,564. There
are five pages of advertisements in the report.
Stockport Infirmary.?Although the average daily
occupation of beds in 1916 was slightly higher than in the
previous year, fewer in-patients were treated. This was
caused by the protracted treatment required in many of
the military oases. The ordinary income amounted to
?8,458, and the ordinary expenditure to ?7,434 9s. 6d.
Legacies of ?625 were received. The report states that
" The Hospital Uniform System of Keeping Accounts
has been introduced, and the committee is convinced that
it presents advantages over the old method."
Royal London Ophthalmic Hospital. ?124,095
attendances of out-patients were made in 1916. During
the year 197 soldiers were admitted as in-patients. The
attendances of L.C.C. school children were 13,275. The
hospital had a good year for legacies, which produced
?4,128, and there was a balance to the good on the
income and expenditure account of ?1,778.
Royal Westminster Ophthalmic Hospital.?In view
, of staff difficulties the number of new out-patients seen
daily is now limited to forty. There was a falling off
in both in- arid out-patients last year, but the attendances
of the latter increased. Total income, ?4,232; total
expenditure, ?4,228. We notice that a gift made by the
trustees of the Zunz Bequest is shown as a legacy;
this is contrary to King Edward's Fund regulations, and
the amount should be included in donations.
Bideford and District Hospital.?The accounts are
net arranged under the Uniform System. The total
ordinary income was ?1,034, and the total ordinary
expenditure ?1,222. Some gifts in kind were received,
but are apparently not valued for the purposes of the
accounts.
Isis District Hospital.?This hospital is situated at
Childers, in Queensland. The average daily bed occu-
pation in 1916 was 4.9, and nineteen outdoor patients
were treated. The activities of the hospital increase or
lessen according to the nature of the harvest. As there
was no " crushing " last year the population of the dis-
trict was not added to by the usual number of special
harvest workers. -
Birmingham and Midland Hospital for Women
and Birmingham Lying-in Charity.?The ordinary
expenditure of the hospital exceeded the ordinary income
by ?1,837. The maintenance fund, which records extra-
ordinary income and expenditure, shows a balance on the
right side of ?611. The Lying-in Charity had an income
of ?5,479, and an expenditure of ?5,780.
Glasgow District' Mental Hospital.?There were
760 patients at the end of the statistical year. " The effect
of the war on our admissions," states the medical super-
intendent, " is not marked in a direct way. A few
weaklings have had attacks determined by excitement in
connection with Army Service, and others have broken
down under the strain of Service conditions. Most of
these would probably have sooner or later broken down
at any rate, and few have seen any active service."
Marine Society. ?Last year 135 boys joined the Royal
Navy and eighty-five were drafted to the Merchant Ser-
vice and vessels chartered by the Admiralty. The Society
awarded eight sextants to old boys who had passed as
second mate in the Merchant Service, and three 3words
to those attaining warrant rank in the Royal Navy.
Leeds Sanitary Committee has agreed to maintain ten
beds at the Leeds Maternity Hospital in consideration of
such cases being available when required for complicated
cases recommended by the Medical Officer of Health.
Personalia.
Captain Harold, M.D., R.A.M.C., who when the war
broke out was senior resident medical officer of the Royal
Infirmary, Liverpool, and Fellow and Lecturer in Physi-
ology at Liverpool University, and has since been in
charge of an officers' hospital in Mesopotamia, has now
been promoted Deputy-Assistant Director of Medical Ser-
vices or; the Lahore General Staff.
V20 THE HOSPITAL ' May 12, 1917.
Matrons' and Sisters'
Appointments.
Auxiliary Military Hospital, Mirfield, Yorks.?Miss
Gertrude E. Sharpe has been appointed matron. She was
trained at the Royal Infirmary, Sunderland, where she was
subsequently sister.
Launceston Infirmary.?Miss Sarah E. Duncan Neil has
been appointed matron. She was trained at Townley's
Hospitals, Bolton, and after a period of work as Queen's
nurse became superintendent nurse of Skipton Infirmary,
and was later appointed matron of Ashton-under-Lyne Red
Cross Hospital.
Kensington Infirmary.?Miss Mabel Turner has been ap-
pointed superintendent of night nurses. She was trained at
the Cardiff Union Hospital, becoming ward sister there after
her training. She then went as ward sister first to Shirley
Warren Union Hospital, and later to the City of West-
minster Union Infirmary, Fulham Road, returning to Car-
diff Union Hospital as night sister.
County and City of Perth Royal Infirmary.?Miss A. S.
Morriss has been appointed sister. She was trained at
the West Kent General Hospital, Maidstone, and has
recently been sister at the Welsh Metropolitan War Hos-
pital, Whitchurch, Cardiff.
Hospital for Officers, 53 Cadogan Square.?Miss Pringle
has been appointed night sister. She was trained at the
Radcliffe Infirmary, Oxford, and has since served with
Lady Minto's Indian Nursing Association, and done military
nursing in France.
Hospital of St. Cross, Rugby.?Miss Fannie Morgan has
been appointed sister. She was trained at the General
Hospital, Wolverhampton, after having spent two years at
the Cripple Children's Hospital, Northfield. Since her
training ehe has been night sister at the General Hospital,
Weston-super-Mare. -
NOTE,?Particulars of Appointments lor insertion in this
column will be welcomed.
Presentation.
During the past week Miss Herbert, Matron
of Worcester General Infirmary, was the re-
cipient of many gifts on her retirement. The sisters,
past, and present nurses, and the domestic staff pre-
sented a Worcester china coffee service and a flower bowl
on an ebony stand. These were accompanied by a letter
signed by eighty-seven members or past members of the
staff, and a subscription to the Matron's Mortuary Fund,
in which Miss Herbert has taken a deep interest. The
letter expressed the high esteem in which Miss Herbert
was held by the staff and their appreciation of her
kindness and courtesy/ On the previous Sunday the
members of the Worcester Cathedral and Infirmary Choir
gave Miss Herbert a leather-bound copy of " Hymns,
Ancient and Modern," inscribed, and containing photo-
graphs of the cathedral and infirmary. In a Tetter from
the Rev. T. C. de la Hey?who before going on active
servioe was a Minor Canon in charge of the choir?which
was read by Mr. W. W. Harris, the conductor, Mr.
de la Hey said Miss Herbert had always shown kindness,
sympathy, and courtesy towards the choir in the work
they had been carrying out for the infirmary by giving
Sunday afternoon services of singing to the patients.
These were begun in February 1893, shortly before Miss
Herbert became matron, and their visits had been made
the more enjoyable by her welcome and encouragement.
The gift was a testimony of their gratitude and goodwill
towards her for many kindnesses. (See also p. 108.)
Gifts and Bequests.
A large motor ambulance has been presented to Goole
Bartholomew Hospital by Mr. and Mrs. Alfred Calvert.
Mr. John Charles Froyne, of Pembroke, formerly
Chief Constructor of H.M. dockyard at Pembroke Dock,
who died on January 4 last, left ?150 to the Pembroke
Dock and District Infirmary.
," In affectionate remembrance of their dear aunt," the
late Mrs. Jeanette Phillips, of St. David's Villa, Uplands,
Swansea, Mr. and Mrs. Christopher Pond, of Bloom-
field House, Blackwood, have given ?1,000 to endow &
"Jeanette Phillips" cot in Swansea Hospital.
A number of Carlisle charities benefit under the will
of Mr. Robert Barton, of The Crescent, Carlisle, among
the bequests being the following : Cumberland Infirmary*
?2,COO; Carlisle Dispensary, ?200; Carlisle District
Nursing Association, ?200; Home for Incurables, Car-
lisle, ?2C0; St. Mary's Home, Carlisle, ?200; Con-
valescent Institution, Silloth, ?200; Blind Asylum,
Carlisle, ?100; and Carlisle Day Nursing Association,
?100.
Annual Meetings.
Asylum Workers' Association.?Monday, May 14,
3 p.m., at the Mansion House, London, E.C., the Right
Hon. the Lord Mayor (Vice-President of the Association)
in the chair.
Miller General Hospital for South-east London,
Greenwich Road, S.E.?Tuesday, May 15, 5 p.m., in the
board-room. (Tea and coffee, 4.30. The hospital, includ-
ing the Almeric Paget massage and electrical department,
will be open for inspection.)
*tt?s oi Subscription : Twelve months, 6/6; Six months, 4/-; Three months, 2/-; post free to any address in the United Kingdom.
Abroad, Twelve months, 8/8; Six months, 5/-; Three months, 2/6, post free. Thi Hospital "Index" to Volume LX., April
to September 1916, is now ready, prioe 2Jd. per oopy, post free. Address The Manager, " Thi Hospital," The Hospital
Building, 28/29 Southampton Street, Strand, London, W.O. 2.

				

